# Hearing what isn't said

**DOI:** 10.1002/ueg2.12644

**Published:** 2024-11-01

**Authors:** Yasuko Maeda

**Affiliations:** ^1^ Department of General Surgery Department of Surgery and Colorectal Surgery Queen Elizabeth University Hospital Glasgow UK; ^2^ University of Glasgow Glasgow UK

Patient care is the cornerstone of our daily clinical practice, yet interest in patients' perspectives of treatment has not received much attention to date. The paper by Dekkers et al.^1^ is a welcome addition to this field. The study attempted to tease out aspects of information giving, decision‐making, and whether the patient was satisfied with the outcome.

The management of early colorectal cancer is becoming increasingly complex with evolving treatment options. This study focused on the option of local excision and follow‐up, including additional treatment. It eloquently describes the need for tweaking information according to patients' understanding, highlights the necessity of nuanced discussion, and identifies areas of communication that could be improved in the backdrop of uncertainties.

The authors have used different scoring systems to explore these aspects. Several quality‐of‐life scales provide quantitative data pertinent to research studies, particularly for comparison. However, quality of life is variable, depending on what matters most to each individual, and it may be influenced by societal values, religious beliefs, and socioeconomic circumstances. The educational level may indicate that the person has obtained a certain level of knowledge and skills to navigate complexities in life. Still, it should not serve as a benchmark of each individual's thirst for knowledge or ability to seek answers to their inquiries to make an informed decision. We must be cautious about simplifying a complex issue into a dichotomizing variable. Otherwise, a nuanced approach will soon become an all‐or‐nothing conclusion.

The study also opens up potential complex issues. Presumably, the minority but existing population who could not read Dutch were excluded, and there was no patient in this cohort with recurrence. This is understandable in the context of research yet highlights the issue of equity and accessibility of information. Do patients get selective discussion when things may be lost in translation, or will clinicians find it difficult to pitch the level of discussion if patients were educated in different schooling systems? Do patients with recurrence regret their decision‐making or feel “misinformed”?

This is not the first study to explore decision‐making.^2^ Both clinicians and patients share the same theme, such as anxieties and uncertainties, yet their feelings about anxieties may well be different. Patients are anxious about whether the treatment is optimal and want to know about follow‐up, while clinicians worry about whether the patients fully understand the information they give.

The ultimate choice of management may be based on more than solid evidence data but could be preference‐sensitive. The key is how information is relayed and how both sides deepen interpretation. As the authors stated, it is pivotal to provide information that is comprehensive and understandable. This is crucial but a significant challenge in this day and age when new data of variable quality and quantity emerges quickly, potentially interlaced with an abundance of misinformation on the Internet. Even for a seasoned clinician, it is a time‐consuming and summonable task to comprehend and contextualize information as new research emerges. However, this is where we clinicians are needed more than ever to make essential information as transparent and understandable as ever to laypeople. In the era of mass data science and AI, what we are attempting here may be quite fundamental in human activity: consideration for others and understanding each other, which is the foundation of communication yet is often so lost in the tsunami of numerical.

## Data Availability

Data sharing is not applicable to this article as no new data were created or analyzed in this study.
